# Non-Celiac Gluten/Wheat Sensitivity—State of the Art: A Five-Year Narrative Review

**DOI:** 10.3390/nu17020220

**Published:** 2025-01-08

**Authors:** Francesca Manza, Lisa Lungaro, Anna Costanzini, Fabio Caputo, Antonio Carroccio, Pasquale Mansueto, Aurelio Seidita, Suneil A. Raju, Umberto Volta, Roberto De Giorgio, David S. Sanders, Giacomo Caio

**Affiliations:** 1 Department of Translational Medicine, University of Ferrara, 44121 Ferrara, Italy; mnzfnc@unife.it (F.M.); anna.costanzini@unife.it (A.C.); fabio.caputo@unife.it (F.C.); dgrrrt@unife.it (R.D.G.); caigmp@unife.it (G.C.); 2 Academic Unit of Gastroenterology, Sheffield Teaching Hospitals NHS Foundation Trust, Sheffield S5 7AT, UK; suneilraju@gmail.com (S.A.R.); david.sanders1@nhs.net (D.S.S.); 3 Unit of Internal Medicine, “V. Cervello” Hospital, Ospedali Riuniti “Villa Sofia-Cervello”, 90146 Palermo, Italy; antonio.carroccio@unipa.it (A.C.); aurelio.seidita@unipa.it (A.S.); 4 Department of Health Promotion Sciences, Maternal and Infant Care, Internal Medicine and Medical Specialties (PROMISE), University of Palermo, 90127 Palermo, Italy; pasquale.mansueto@unipa.it; 5 Institute for Biomedical Research and Innovation (IRIB), National Research Council (CNR), 90146 Palermo, Italy; 6 Department of Medical and Surgical Sciences, University of Bologna, 40138 Bologna, Italy; umberto.volta@unibo.it; 7 Mucosal Immunology and Biology Research Center, Massachusetts General Hospital—Harvard Medical School, Boston, MA 02114, USA

**Keywords:** non-celiac gluten sensitivity, non-celiac gluten/wheat sensitivity, NCGWS, gluten, gluten-free diet

## Abstract

**Background:** Non-celiac gluten/wheat sensitivity (NCGWS) is a syndrome for which pathogenesis and management remain debated. It is described as a condition characterized by gastrointestinal and extra-intestinal symptoms rapidly occurring after gluten ingestion in subjects who have had celiac disease or wheat allergy excluded. To date, the diagnosis of NCGWS is challenging as no universally recognized biomarkers have been yet identified, nor has a predisposing genetic profile been described. However, the research is moving fast, and new data regarding pathogenic pathways, patients’ classification, potential candidate biomarkers, and dietary interventions are emerging. **Methods:** This literature review aims to address the state of the art and summarize the latest updates in this field from 2019 to date. **Results and Conclusions:** Clinical studies regarding NCGWS in the last five years are reported to shed light on this complex condition and to guide specialists towards a more in-depth, prompt, and objective diagnosis.

## 1. Introduction

Non-celiac gluten sensitivity (NCGS) is a syndrome for which the pathogenesis and management are still debated. It is described as a condition in which intestinal and extra-intestinal symptoms occur after gluten/wheat ingestion in subjects where the diagnosis of both celiac disease (CD) or IgE-mediated wheat allergy (WA) has been excluded. To date, a genetic background predisposing to NCGS has not been identified, and unlike CD and WA, diagnostic biomarkers for NCGS diagnosis are lacking. Wheat is a complex mixture of proteins, starches, fibers, and micronutrients, cheap and widely available. The term gluten refers to a family of storage proteins (prolamins) contained in wheat, rye, barley and their cross-bred grains [1,2], and its proline- and glutamine-rich residues, gliadins, are the trigger for CD in genetically predisposed subjects. Meanwhile, for WA, the proteins detected as a trigger for the development of an IgE-mediated response are lipid transfer protein (LTP, Tri a 14), omega-5-gliadin (Tri a 19) and the amylase trypsin inhibitor family (ATI).

The mechanisms involved in NCGS remain only partially understood, with some aspects of immunity likely contributing to its clinical manifestations. Symptoms can be triggered by gluten as well as by other wheat components, such as those contained in fermentable oligosaccharides, disaccharides, monosaccharides, and polyols (FODMAPs); ATIs, wheat germ agglutinin (WGA); and wheat glyphosate [1]. ATIs are highly resistant to intestinal proteolytic degradation and have been identified as strong activators of innate immune responses in human and murine macrophages, monocytes, and DCs, eliciting the release of pro-inflammatory cytokines via the activation of TLR4 [3]. WGA induces the activation of phlogistic pathways and epithelial barrier disruption [3,4]. However, more in vivo human studies are needed to better clarify their role in NCGS. Based on this evidence, in this review, we elected to use the more inclusive term “non-celiac gluten/wheat sensitivity (NCGWS)” to encase all of the components leading to symptoms onset.

The innate immune response is believed to play a key role in the pathology of NCGWS through an increased expression of Toll-like receptors and an imbalance between regulatory and pro-inflammatory cytokines. Other factors that may contribute to the development of symptoms are intestinal barrier impairment and microbiota alteration (i.e., dysbiosis). These are known to cause a slight increase in intraepithelial lymphocytes, mast cells, and eosinophils while preserving villous architecture [5,6].

NCGWS includes a wide array of symptoms; abdominal pain, bloating, reflux, and irregular bowel movements are the most common gastrointestinal (GI) symptoms, but a systemic involvement has often been reported, including fatigue, headache, “foggy mind”, and dermatitis [7]. Given the significant overlap in symptoms with irritable bowel syndrome (IBS), NCGWS can be difficult to diagnose. The definition of NCGWS has thus far relied on:

(1) Exclusion of CD and wheat allergy;
(2) The patient’s responsiveness to a gluten-free diet (GFD);
(3) Double-blind, placebo-controlled gluten/wheat rechallenge, according to the “Salerno experts’ criteria” [5,8].

The treatment currently consists of a gluten-free diet (GFD) that resolves both intestinal and extra-intestinal symptoms, albeit without being as strict as required for patients with CD. The only guideline for the management of NCGWS can be found in the “European Society for the Study of Coeliac Disease (ESsCD) guideline for CD and other gluten-related disorders” [9].

In this review, recent clinical trials will be discussed to update physicians managing patients with NCGWS. The lack of biomarkers, similarity of symptom profile to other GI conditions, and the limited treatment options will be considered along with directions for future research.

## 2. Materials and Methods

This narrative review aims to describe the updated state of the art of NCGWS in the last five years. Two independent authors (F.M. and L.L.) performed a comprehensive and independent literature search on PubMed, MEDLINE, and ScienceDirect using the following search terms in the title and abstract: “Non-celiac gluten/wheat sensitivity”; “Non-celiac Wheat Sensitivity”; and “Non-celiac Gluten Sensitivity”, operators “AND” and “OR”. Only articles (1) consistent with the topic; (2) with an original randomized controlled trial (RCT); (3) published in the last five years, from January 2019 to October 2024; (4) written in English; (5) listed as research articles; and (6) available in full text were included in this narrative analysis. Related results fitting the research criteria were also considered. Records were subsequently reviewed by the other authors. Any disagreements between F.M. and L.L. were resolved by consensus with G.C. and R.D.G. Three papers have been excluded because they were not relevant for the aims of this review [10,11,12]. Articles not addressing the inclusion criteria, duplicates, and incomplete articles or those with unclear outcomes were excluded from this review.

## 3. Latest Clinical Trials (2019 to 2024)

**Figure 1:**
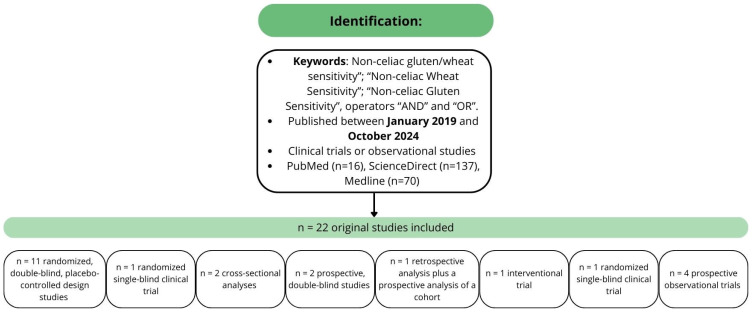
Methodology and studies characteristics.

From 2019 to September 2024, twenty-two original clinical trial studies regarding NCGWS were published. Eleven were randomized, double-blind, placebo-controlled design studies [13,14,15,16,17,18,19,20,21,22,23], one was a randomized single-blind clinical trial [24], two were cross-sectional analyses [25,26], two were prospective, double-blind studies [27,28], one was a retrospective analysis plus a prospective analysis of a cohort [29], one was an interventional trial [30], and finally, four were prospective observational trials [11,31,32,33,34]. The details of studies included in this review have been reported in Section 3.1, Section 3.2, Section 3.3, Section 3.4 and Section 3.5 and summarized in Figure 1.

### 3.1. Pathogenetic Mechanisms

To date, the pathogenesis of NCGWS has not been sufficiently investigated. The only study analyzing risk factors for the development of NCGWS is by Gambino et al. [25]. They analyzed the role of killer immunoglobulin-like receptors (KIRs) genes and haplotypes as predisposing factors for the development of NCGWS. KIRs are surface receptors specific for allelic forms of human leukocyte antigen (HLA) class I molecules, a highly polymorphic family of genes. In CD, KIRs regulate the natural killer cells (NKs) response, and they can be classified as having an activating or inhibitory activity. The authors compared patients with CD, NCGWS, and healthy controls. They found a decreased frequency of KIR2DL1, -2DL3, -2DL5, -2DS2, -2DS3, -2DS4, -2DS5, and -3DS1 genes and an increased frequency of -3DL1 gene in the NCGWS cohort, leading to the authors hypothesizing their involvement in NCGWS susceptibility, with KIR2DL5, -2DS4, and -2DS5 having a protective effect. Using confocal laser endomicroscopy (CLE), Fritscher-Ravens et al. [16] showed a non-IgE-mediated mechanism, triggering a mucosal response in patients with IBS symptoms. CLE patients with NCGWS were found to generate a local response (CLE+), characterized by an increase in intraepithelial lymphocytes, mucosal leaks, and intercellular extravasation of fluorescein-labeled plasma fluid into the widening intervillous space upon exposure to wheat. Moreover, the duodenal fluid had significantly higher concentrations of eosinophilic cationic protein (ECP), a higher expression of claudin-2, and lower levels of occludin. The withdrawal of food components causing a CLE+ reaction, including wheat, reduced IBS symptoms in CLE+ patients. Although more research is needed in this field, the study by Fritscher-Ravens et al. paved the way to a better understanding of the mucosal mechanism related to food ingestion and the subsequent generation of symptoms in predisposed patients.

Table 1 briefly summarizes the details of these studies.

#### Summary for Pathogenetic Mechanisms

To date, the pathogenesis of NCGWS remains unclear. Therefore, the role of genetic risk factors should be further investigated with larger cohort studies.

The presence of non-IgE-mediated reactions to wheat in patients with IBS symptoms suggests the existence of pathological mechanisms involving a response to wheat proteins (including gluten, non-gluten-related protein, and ATIs), although further research is still needed.

### 3.2. Clinical Features and Correlation of Symptoms with Specific Food Components’ Ingestions

The spectrum of NCGWS symptoms encompasses both GI (e.g., bloating, abdominal discomfort and pain, diarrhea, and flatulence) and extra-GI manifestations (tiredness, headache, and anxiety) [35]. Moreover, other sensory symptoms, such as tingling and numbness, have been described by Hadjivassiliou et al. and defined as responsive to gluten withdrawal in a cohort of patients with gluten sensitivity [36].

More recently, nine studies analyzed the clinical features and correlation with certain nutrients in patients with NCGWS [14,17,18,20,22,26,29,31,34].

Skodje et al. [26] registered the dietary intake of 65 self-reported NCGWS patients and examined clinical symptoms and health-related quality of life (HR-QoL). In this cohort, CD and WA were excluded, and patients had been on a GFD for at least six months. Eighty-eight percent of the population were female, and participants had a lower HR-QoL than the general population. Moreover, the consumption of total and saturated fat was higher and the intake of carbohydrate and dietary fiber was lower than the recommended daily amount. Moreover vitamin D, folic acid, calcium, iodine, and iron assumptions were not in the recommended range, but blood values did not show nutrient deficiencies. The adherence to a GFD and a mean moderate–low intake of FODMAPs (11.6 g) did not significantly reduce intestinal symptoms. These data are part of an RCT carried out by the same authors [37], where a challenge with fructans, rather than gluten, showed a significant worsening of GI symptoms. However, there are limitations in this study as the recruited cohort self-reported gluten intolerance and they were not diagnosed with the gold standard method of a double-blind placebo-controlled assessment (DPBC-C). Thus, some of them might represent a sub-population of patients with IBS or small intestinal bacterial overgrowth (SIBO). Also, Skodje et al. did not report other extra-GI symptoms and immune-mediated diseases, apart from thyroiditis.

Moleski et al. [14] recruited patients with self-reported gluten intolerance and compared them with a group of healthy subjects on a GFD. Participants received pills containing 0.5 g or 2 g of gluten/day for 7 days or placebo. No significant worsening in symptoms after gluten ingestion was observed in both groups. Of note, the patients with NCGWS did not have a diagnosis confirmed by a clinician, and the consumption of other dietary components was not studied. Furthermore, for the same reasons explained above, we cannot exclude the presence of a selection bias in the study population (inclusion of IBS or SIBO patients).

Barone et al. [17] evaluated NCGWS in patients with previous diagnosis of IBS. The authors analyzed the change in visual analog scale (VAS) modification at five timepoints in a FODMAPs-gluten-containing diet (two weeks, t0), after a low FODMAPs-GFD (two weeks, t1), and only patients presenting an improvement in symptoms after a DPBC-C with gluten with wash-out and crossover (t2, t3, t4, one week each). Using the method established by Catassi et al. [8], who considered a significant response a reduction of ≥30% in VAS score, 12 out of 26 patients could fit the diagnosis of NCGWS. However, when considering a VAS score variation greater than the mean ∆-VAS score by +2 standard deviations, as performed by Di Sabatino et al. [38], only five patients were identified as NCGWS. This study aimed to highlight a possible confounding role of FODMAPs in the identification of NCGWS; the authors concluded that FODMAPs intolerance could hide the response to a challenge test with gluten, and therefore, a low FODMAPs-GFD followed by gluten/placebo challenge could identify better patients with NCGWS.

Focusing on different aspects, Herfindal et al. [22] investigated the role of fructo-oligosaccharides (FOSs or fructans) in the intestinal microbial composition and GI symptoms onset in self-reported NCGWS patients. The authors carried out a 7-day-long crossover challenge with gluten-containing, FOS-containing, and placebo bars and collected IBS-related symptoms via the Gastrointestinal Symptom Rating Scale (GSRS-IBS) questionnaire and stool samples. Alpha and beta diversity, fecal metabolites (short-chain fatty acids, SCFAs), and fecal neutrophil gelatinase-associated lipocalin 2 (NGAL/LCN2) did not change across diet challenges, but the relative abundance of certain bacteria taxa were affected. After the FOS-fructans challenge, Fusicatenibacter increased, whereas Eubacterium (E.) coprostanoligenes group, Anaerotruncus, and unknown Ruminococcaceae genera decreased. The gluten challenge was primarily characterized by an increased abundance of Eubacterium (E.) xylanophilum group. However, the statistical association between the variation of certain taxa and GI symptoms showed only a few significant associations. The authors highlighted how the reduction in E. coprostanoligenes group following the FOS-fructans challenge was associated with increased abdominal pain. The heterogeneity of the group of patients included in the study and the short length of the challenges (7 days) are probably the most consistent limitations in this original and innovative study. A longer duration of the crossover challenge could have led to more consistent changes in microbial composition and fecal metabolites [39]; however, the effort requested of patients would have been higher.

Cotton et al. [31] assessed QoL and sleep quality in patients with self-reported NCGWS compared with CD following a GFD. Patients with NCGWS adhered to a GFD less often than those with CD. Furthermore, lower rates of adherence to a GFD in patients with NCGWS were associated with a poorer QoL and worse sleep performance. This study gives interesting insights regarding the application of a GFD in NCGWS patients: patients with NCGWS who adhere to a GFD had better QoL and sleep than those continuing to eat gluten. There is little support available to this group of patients, and there is scarce evidence regarding the benefits/risks of following a lifelong GFD, and while there is evidence to support clinicians in recommending a GFD to NCGWS patients, it is not mandatory for the diet to be as strict as for CD, often identifying a personal threshold for gluten tolerance [9].

GI tract dysmotility has been described in CD [40,41] and to a lesser extent in NCGWS [42]. Two studies examined NCGWS and functional dyspepsia (FD) [18,20]. Shahbazkhani et al. [18] evaluated the presence of refractory FD after a DPBC-C with gluten. Out of 27 patients, 5 (18.5%) were diagnosed with NCGWS after challenge. Four were female, and a high titer of anti-gliadin antibodies (AGAs) IgG was only found in one subject. Potter et al. [20] considered FODMAPs (fructans in particular) in their double-blind challenge. Interestingly, their study design was modified by the Salerno criteria: patients on a normal wheat-containing diet were instructed to follow a low FODMAPs-GFD for 4 weeks (run-in). Subjects with a significant reduction of symptoms underwent a rechallenge using either fructan-containing, gluten-containing, or placebo bars. Unfortunately, due to under-recruitment (only 11 patients enrolled and 5 stopped during run-in phase), a dietary rechallenge was not tried. However, the study highlighted a trend towards the improvement of dyspeptic symptoms on a low FODMAPs-GFD. These two studies are in line with the findings of Elli et al. (2016) [43], where a subset of patients with FD positively responded to a GFD approach. Further research is required to establish the role of gluten exclusion in patients with FD and whether it could be used after first-line treatments have failed.

More extensively, Cobos-Quevado et al. [34] have studied the whole GI transit time in both newly diagnosed CD and NCGWS patients, diagnosed with a gluten challenge, using a wireless motility and pH capsule (WMC). Patients with NCGWS showed improvements in intestinal transit time and contractility when on GFD, although the colon exhibited no discernible effect. The GFD did not significantly impact intragastric, intestinal, or colonic pH; however, given the improved transit time, similar to in CD, inflammation and epithelial alterations associated with intestinal motor dysfunctions may occur in patients with NCGWS. However, results should be interpreted cautiously as the sample size is small (CD = 12, NCGWS = 12) and there is an absence of a control group.

Mansueto et al. [29] analyzed the frequency, severity, and morphologic characteristics of anemia in a cohort of 244 NCGWS patients compared with IBS and CD patients. At the time of diagnosis, patients having IBS-like, dyspepsia-like, and extra-intestinal symptoms were also frequent. Eighty-five participants had anemia (all females; frequency of anemia of 34.8% in NCGWS vs. 17.4% in IBS, *p*=0.03, vs. 48,3% in CD, not significant). NCGWS patients with anemia showed iron deficiency more frequently than non-anemic patients and higher TSH levels. Of these 85 patients, 31 were re-evaluated after at least 12 months of wheat-free diet. A statistically significant improvement in hemoglobin values, mean corpuscular volume, mean corpuscular hemoglobin, and ferritin levels was found. Different pathogenetic mechanisms may account for anemia in this setting, including: (1) iron plus folic acid and/or vitamin B12 deficiency; (2) hypothyroidism; (3) poly/hypermenorrhea (detected in 43% of female patients); and (4) diagnostic delay that might increase noxious effect of wheat in sensitive patients.

Table 2 summarizes the details of these studies.

#### Summary for Clinical Features and Correlation of Symptoms with Specific Food Components’ Ingestions

- Along with classical gastrointestinal symptoms (e.g., bloating, abdominal pain, flatulence) systemic manifestation such as fatigue, tiredness, neurological manifestation, can also be presented symptoms in patients with suspect NCGWS;
- To avoid conflicting results between studies, unambiguous criteria should be used to enroll patients (e.g., Salerno criteria vs. self-reported NCGWS);
- The role of FODMAPs vs. gluten in the development of symptoms is still debated;
- The application of a GFD (regardless of fructans content) seems to be beneficial in the management of symptoms and QoL in NCGWS patients;
- A GFD might be considered an appropriate treatment in a subset of FD patients;
- The presence of an impaired GI motility in NCGWS patients needs to be further assessed;
- NCGWS shares its clinical presentations with several other conditions, including IBS and some neurological/psychiatric conditions. However, the application of a GFD as a treatment option should be considered on a case-by-case basis after discussion with the patient. The effect of the GFD on symptoms should subsequently be reviewed, and the nutritional status of the patient also monitored.

### 3.3. Diagnostic Tools

To date, a diagnostic biomarker has not been identified yet, and the diagnosis of NCGWS currently relies on a DPBC-C, which is often very difficult to carry out in daily practice. The identification of a reliable diagnostic tool would be of paramount importance for patient identification and for a better understanding of causal agents.

Barbaro et al. [32] evaluated serum zonulin as a candidate biomarker. Zonulin is a single-chain protein that is able to reversibly open tight junctions. Higher serum zonulin correlates with increased epithelial permeability, and gliadin can increase its release [44]. Tight junctions opening increases intestinal permeability, a trigger mechanism involved in the initiation of CD and NCGWS. In Barbaro et al.’s study, CD and NCGWS patients showed significantly increased zonulin levels as compared with patients with diarrhea-predominant IBS (IBS-D) and asymptomatic controls. Zonulin levels were reduced after a 6-month wheat-free diet (WFD) only in HLA-DQ2/8-positive participants with NCGWS. The diagnostic accuracy of zonulin levels in distinguishing NCGWS from IBS-D was 81%. The authors also proposed a diagnostic strategy combining gender, zonulin levels, and symptoms that could improve the accuracy up to 89%. However, a possible limitation of this study is the increased serum zonulin levels that may occur in genetically predisposed, completely asymptomatic individuals.

An alternative method proposed by Bojarski et al. [27] tried to develop a diagnostic test for wheat sensitivity in IBS patients. They used CLE with duodenal antigen (wheat, yeast, milk, soy) provocation. CLE generated high-resolution images of the duodenum and was able to detect fluorescein leakage (major criterion) to identify an increase in intraepithelial lymphocytes (IELs) and variations in intervillous spaces (minor criteria). CLE testing was considered positive if at least one major and one minor criterion were documented after wheat administration (CLE+). Patients were then instructed to follow a GFD and register symptoms. Overall, patients with IBS who were CLE+ after application of wheat were approximately twice as likely to have wheat sensitivity compared with those who were CLE-negative after wheat exposure. However, due to its invasiveness and low sensitivity and specificity for wheat sensitivity (less than 80%), CLE is not recommendable as a diagnostic test.

Seidita et al. [28] have proposed an already well-established test in the assessment of NCGWS, i.e., measuring fecal calprotectin (FCP) to identify the presence of an inflammatory status in NCGWS as an index differentiating NCGWS from IBS/FD. They enrolled 201 NCGWS patients (diagnosed with a DPBC-C) and 50 IBS/FD patients. Among patients with NCWS, 31.3% (63/201) had increased FCP values (NCGWS FCP+), whereas all IBS/FD patients had values within the normal range. In the prospective phase of the study, the effects of a strict GFD for 6 months in the NCGWS cohort were assessed. With respect to NCGWS FCP+ group, 65.1% of participants had negative values of FCP after 6 months. The authors concluded the presence of two NCGWS subgroups: NCGWS FCP+, characterized by a predominantly inflammatory/immunologic pattern, and NCGWS FCP-, featuring non-immuno-mediated etiopathogenetic mechanisms. It is difficult to establish the significance of this difference, which may be due to multiple pathological processes, or even address separate disease entities.

Another study focused on the assessment of a helpful tool for distinguishing between NCGWS and IBS [33]. As previously suggested [45], AGA IgAs are present in around half of patients with NCGWS. On this assumption, the authors enrolled 492 patients with IBS: those who were AGA (IgA or IgG)-positive (61, 12.4%) were asked to follow a GFD for 6 weeks. Patients who had an improvement in symptoms were rechallenged with gluten. Of the 31 patients who agreed to follow a GFD, 17 (54.8%) had complete (>30% improvement) and 10 (32.2%) had partial (20–30% improvement) responses. Despite the small cohort, AGA might be further explored as a helpful tool for differential diagnosis for NCGWS, at least for identifying a subset of patients.

See Table 3 for a summary of these studies.

#### Summary for Diagnostic Tools

- To date, no candidate biomarkers or diagnostic tool have shown an adequate reliability for diagnosing NCGWS;
- Further research is required to assess the role of additional tests (zonulin, FCP, AGA) in the diagnosis of NCGWS.

### 3.4. Dietary Interventions

Five studies evaluated different dietary interventions and their consequences on several aspects of NCGWS [15,19,21,24,30].

Ajamian et al. [13] evaluated the effects of FODMAPs and gluten on markers of intestinal epithelial injury (syndecan-1 and intestinal fatty acid-binding protein) and bacterial translocation (lipopolysaccharide-binding protein and soluble CD14). The cohort included patients with IBS and self-reported NCGWS. Only syndecan-1 decreased in a low FODMAPs diet along with a reduction in symptoms, regardless of the presence of gluten. The authors concluded that epithelial integrity and symptoms are not affected by gluten ingestion in their study population; however, patients did not undergo a DPBC-C to confirm a NCGWS diagnosis.

Roncoroni et al. [30] exposed NCGWS patients to increasing amounts of gluten in an unblinded fashion: 3.5–4 g/day for week 1, 6.7–8 g/day for week 2, and 10–13 g/day for week 3. Patients were diagnosed according to the Salerno criteria. Among the 24 patients enrolled, 8 did not tolerate even a low gluten content, 6 relapsed on diet containing 6.7/8 g of gluten/day, and 8 tolerated a higher gluten containing diet. This study shows that there is a certain level of tolerance in NCGWS patients, and therefore, a controlled reintroduction of gluten might be helpful for improving the QoL in a specific group of patients.

Ianiro et al. [19] tested, in a DPBC-C crossover design study, Senatore Cappelli (a variety of wheat) in a cohort of NCGWS patients. Senatore Cappelli has shown more favorable characteristics, such as higher content of fibers and micronutrients and a reduced gliadin content and pesticide contamination [46]. Significantly lower overall and GI symptoms scores, as measured by GSRS, were reported in the study group eating Senatore Cappelli pasta compared with the one eating standard pasta. This might support the application of a less strict GFD for patients with NCGWS and the presence of dietary alternatives (such as ancient wheats with a lower content of gliadin and pesticides) with consequent health, economic, and social benefits.

Zimmermann et al. [24] evaluated the tolerance of spelt vs. wheat in patients who referred wheat intolerance and simultaneous spelt tolerance, assessed before the beginning of the trial. They used six types of bread: a gluten-free (GF) bread, bread supplemented with gluten or FODMAPs, and spelt or wheat bread both baked accordingly to “traditional” (T) or “current” (C) recipe. The IBS-Severity Scoring System (IBS-SSS) questionnaire was used to assess GI symptoms severity. IBS-SSS scores were higher than expected by the participants after spelt bread consumption and lower for wheat bread consumption, resulting in no difference between spelt and wheat bread tolerance. The results highlighted a high prevalence of a nocebo response (40% of patients). Markers for intestinal permeability (serum zonulin and lipopolysaccharide-binding protein) did not change between the different breads. However, in their study design, a shorter challenge and wash-out between the breads was used (challenge 4 days, wash-out 3 days) compared with other trials that usually performed longer challenges and wash-out periods (most commonly lasting a week) [37,38].

Another study evaluated the effect of different types of wheat and fermentation techniques [23]. Patients with self-reported NCGWS were asked to adhere to a “symptom-free diet” (replace or avoid food products that they considered to induce GI symptoms). Subsequently, they received either fermented yeast (FY) bread composed of wheat, spelt, or emmer (group A), or fermented sourdough (FS) bread, also composed of wheat, spelt, or emmer (group B). It is believed that sourdough fermentation leads to fructan degradation and improves digestive tolerance. Moreover, the content of gluten in spelt and emmer is reduced by 20% compared with wheat. Therefore, the authors hypothesized that consumption of FY and FS bread composed of emmer and spelt may cause less GI and extra-GI symptoms than conventional bread. Interestingly, no differences were found when comparing wheat or fermentation type, but the authors highlighted how more than 50% of the participants developed GI symptoms to more than one type of bread. It must be noted that all bread types contained FODMAP, gluten, and ATIs: for these reasons, assigning any of the reported symptoms to one of these components was not possible. However, the study population was very heterogenous, especially in relation to food choices that relieved/relapsed symptoms caused by wheat ingestion.

Lastly, De Graaf et al. [21] quantified the presence of a nocebo effect in people with self-reported NCGWS. They investigated the effects of expectancy (E) about gluten intake versus actual (G) gluten intake on GI and extra-intestinal symptoms. Eighty-four patients were randomized into four groups: E+G+ (expectancy to consume gluten-containing bread, combined with actual intake of gluten-containing bread); E+G− (expectancy to consume gluten-containing bread, combined with actual intake of gluten-free bread); E-G+ (expectancy to consume gluten-free bread, combined with actual intake of gluten-containing bread); and E-G− (expectancy to consume gluten-free bread, combined with actual intake of gluten-free bread). Their results showed that the combination of positive expectancy and actual gluten intake had the largest effect on overall GI symptoms; moreover, actual gluten intake did not affect overall or individual symptoms. The fact that the E+G+ group had the highest symptoms score might point out a direct involvement of gluten in the worsening of the VAS scores but also a heterogeneity of mechanisms that lead to the development of symptoms.

See Table 4 for a brief summary of these studies.

#### Summary for Dietary Interventions

- NCGWS patients may be able to tolerate wheat to some degree. However, the role of its components (gluten, FODMAPs) remains unclear; focusing on the uniformity of the patients enrolled among different studies might be pivotal for achieving conclusive results;
- The expectancy of wheat ingestion and the presence of a nocebo effect are emerging but do not help distinguish features in NCGWS. Further assessments are required, which may include differentiating patients into different subsets;
- The dietary approach should be tailored to each patient’s preferences and wheat/FODMAPs tolerance.

### 3.5. Therapeutic Strategies

Due to the poor understanding of NCGWS, there are limited therapeutic targets. The literature on therapeutic approaches for treating NCGWS is therefore sparse. The only study analyzing a possible therapeutic strategy was the one by Scricciolo et al. [13]. They assessed the efficacy of a proline-specific endopeptidase enzyme isolated from Aspergillus niger (P1016) with high specificity for the degradation of proline-rich gluten epitopes. Patients assumed either a placebo or capsule containing P1016 in a blinded fashion and were instructed to follow a diet containing increasing amounts of gluten for 21 days. Over the same period, the capsules were administered right before gluten consumption, once a day for the first week, twice a day for the second week, and thrice a day during the third week. Abdominal pain, stool consistency, severity of abdominal swelling, severity of postprandial fullness, severity of early satiety, epigastric burning, state of satisfaction regarding general well-being, and QoL were recorded with VAS score and SF-36 (Short Form health survey-36) scoring. The results did not show any differences in GI and psychological symptoms or QoL using enzyme P1016, despite its ability to break down gluten in vitro. Table 5 briefly summarizes this study.

#### Summary for Therapeutic Strategies

- To date, there are no effective alternative treatments, rather than a dietary approach, for the improvement of GI symptoms and QoL in patients with NCGWS.

## 4. Discussion

Several original studies on NCGWS have been published in the past few years, and all of them have clearly indicated that NCGWS is still a debated clinical entity that is difficult to recognize and manage.

An accurate analysis of the currently available literature leads to the following considerations. First, the lack of understanding of the pathogenesis of NCGWS may affect the patients’ homogeneity and recruitment. Despite the Salerno expert criteria published in 2015, only 13 out of 21 trials here listed [13,17,18,19,20,25,27,28,29,30,32,33,34] recruited or diagnosed NCGWS patients according to DPBC-C. Indeed, DPBC-C is not practical and easy to use in regular clinical practice. As a result, it currently remains as a research tool. Limitations regarding the availability of identical gluten-containing/gluten-free foods to be used for challenge, the inability to discriminate the harmful component(s) involved, and a role for a nocebo effect are all factors further hampering the progress in understanding NCGWS. Moreover, the application of DPBC-C in clinical studies might imply early discontinuation or screening failure due to the inability of the patients to follow the protocol [20]. Therefore, the need for a reliable biomarker for diagnosis is needed. At the same time, the lack of a well-defined pathogenetic pathway hinders the findings of a diagnostic tool.

FODMAPs are known for their osmotic and fermentable properties, which can lead to severe bloating, pain, and diarrhea in a subset of patients, and are considered to be one of the possible culprits for symptom generation in NCGWS. FODMAPs are known to have a role in eliciting symptoms in IBS due to their characteristics, and a low FODMAPs containing diet is recommended for the management of IBS in several guidelines [47,48,49]. Moreover, many NCGWS symptoms overlap with the one reported in IBS, specifically bloating and abdominal pain. Because wheat is one of the main sources of dietary fructans, Skodje et al. [37] highlighted an increase in symptoms in self-reported gluten-sensitive patients, implying a role for this dietary component rather than gluten (or placebo). Fernandes Dias et al. suggested that a complete low FODMAP diet can further decrease GI symptoms in patients with NCGWS [50]. Molina-Infante and Carroccio [51] summarized the challenges derived from the application of the DPBC-C based on gluten administration only for the diagnosis of NCGWS, stating that less than 20% of patients can be confirmed as gluten/wheat sensitive accordingly to the Salerno criteria.

Based on these considerations, a more complete approach for the diagnosis of NCGWS should be the evaluation of the gluten challenge response after the administration of a low FODMAP/GFD diet. This strategy would not only be able to recognize patients with NCGWS, but also IBS patients responsive to a low FODMAP diet vs. patients not responsive to dietary treatments. Unfortunately, a DPBC-C with three arms may turn out to be actually very difficult for physicians and patients.

The presence of a nocebo response during gluten consumption has been reported in several studies, reaching a prevalence of 40% among the participants [21,24]. Therefore, sociological aspects should also be considered when describing NCGWS, specifically the perception of the healthiness of GFD nowadays. The self-reported connection between symptoms and gluten ingestion may limit patient recruitment in clinical trials, and as a consequence, their results are often conflicting.

Around 25% of consumers see gluten-free products to be healthier than their gluten containing counterparts [52]. The lack of appropriate biomarkers has resulted in a self-diagnosed NCGS, and self-reported wheat sensitivity (SRWS) is common in young to middle-aged women who consider wheat-based products to be the culprit for intestinal and extra-GI symptoms. Typical common traits are a previous diagnosis of other GI disorders (i.e., IgE-mediated allergy, multiple food hypersensitivity, IBS) and mood disorders (i.e., anxiety and depression). These patients are usually less receptive to conventional medicine and usually seek alternative treatments in complementary medicine [10].

Notably, in the study by S. Golley et al., the decision to avoid wheat-based products was only supported by a formal medical diagnosis in 5.7% of the analyzed sample [53]. Moreover, more than half of wheat avoiders are also restricting dairy products. Data from Priven et al. [52] underline that “free-from” products generate a perception of healthiness, especially in those that, because of different reasons, perceive gluten as a “risky” food component. The assumption of the harmful properties of gluten often comes from misinformation carried on by media and the Internet; therefore, it is falsely claimed that avoiding gluten increases overall health status.

Another misconception that might lead people to pursue a GFD is that eating gluten-free helps lose weight [54]. However, from the experience of CD patients, studies suggest that body weight is more likely to increase after a GFD regime [55,56]. Even though this might also be related to an increased ability to absorb nutrients, indeed, GFD products are often lower in protein and fiber content and higher in fats, salt, and sugar [57]. In addition, maintaining a GFD lifestyle has many challenges, including nutritional deficiencies, high costs, and social and psychological barriers [58].

Therefore, the term NCGWS does not always seem to include a homogeneous group of patients. As highlighted by De Graaf et al. [21], the combination of expectancy and actual gluten intake had the largest effect on overall GI symptoms. This suggests that a role for gluten itself cannot be excluded but might imply at the same time the involvement of a central processing of external pieces of information (the expectancy) influencing gastrointestinal sensory and vice versa. This bidirectional connection between the GI tract and the nervous system is a distinguishing feature of the disorders of the gut–brain interaction (DGBI), a definition that includes both FD and IBS [59]. Some studies analyzed in this review [18,20,33] suggest an overlap between DGBIs and NCGWS, while others stress more distinctive and organic features of NCGWS [25,28,29]. However, in clinical practice, an accurate distinction between DGBIs and NCGWS can be challenging due to the presence of shared symptoms and the absence of a reliable biomarker. Moreover, the majority of IBS patients believe that certain food items are important triggers of their GI symptoms, specifically high carbohydrate-containing products or histamine-releasing, amine-rich food items [60,61]. Ultimately, a low FODMAPs-containing diet has shown a reduction in clinical and psychological symptoms in NCGWS in several studies [50,62].

What has been summarized here might suggest the presence of at least three subgroups of patients: one with a specific reaction to gluten (antibodies positivity—AGA, genetic background [45,63]), another sensitive to other wheat’s components (ATIs, WGA, FODMAPs, and wheat), and a third belonging to a IBS/FD/DGBI group of patients, implying a multifactorial etiology of NCGWS. However, this hypothesis needs further validation.

## 5. Conclusions

Over the past five years, several original and significant papers on NCGWS have been published, and the most relevant data focus on pathogenesis, clinical features, candidate diagnostic tools, and dietary and therapeutic strategies. The intense research in this field has improved our understanding of NCGWS, although further studies are necessary to improve the knowledge on this debated clinical entity and develop a reliable biomarker for diagnosis and management.

## Figures and Tables

**Table 1:** Studies regarding pathogenetic hints from 2019 to 2024.

Title	Authors	Type of Study	Population	Diagnostic Criteria for NCGWS	Methods	Results	Author’s Conclusions
The role of Killer immunoglobulin-like receptors (KIRs) in the genetic susceptibility to non-celiac wheat sensitivity (NCWS)	Gambino CM. et al., 2024 [25]	Cross-sectional study.	50 NCWS, 50 CD, 50 HCs	DPBC-C	KIR genes and KIR genotyping were investigated with the PCR-SSOP method.	In NCWS, the frequency of KIR2DL1, -2DL3, -2DL5, -2DS2, -2DS3, -2DS4, -2DS5, and -3DS1 genes was decreased. The frequency of -3DL1 gene was increased with respect to both CD and HCs; KIR haplotype expression was similar between the groups. KIR2DL5, -2DS4, and -2DS5 were independent predictors of NCWS.	There might be a role of KIR genes in NCWS susceptibility, with KIR2DL5, -2DS4, and -2DS5 having a protective effect.
Many patients with Irritable Bowel Syndrome have atypical food allergies unrelated to Immunoglobulin E	Fritscher-Ravens et al., 2019 [16]	Prospective randomized clinical trial	108 IBS (Rome III) patients	Not assessed	Patients with IBS received 4 challenges with wheat, milk, soy, yeast, or egg white via the endoscope, followed by CLE. Immunoglobulin E serology analysis and skin tests were used to exclude classical food allergies. Duodenal biopsy samples and fluid were collected 2 weeks before and immediately after CLE. Histology, immunohistochemistry, reverse transcription polymerase chain reaction, and immunoblots were performed. Results from CLE^+^ were compared with results from CLE− or HCs.	76 participants were CLE^+^ (70%), and 46 of these (61%) reacted to wheat; CLE^+^ patients had a 4-fold increase in prevalence of atopic disorders compared with controls (*p* = 0.001). IEL were significantly higher in duodenal biopsy samples from CLE^+^ vs. CLE− patients or controls (*p* = 0.001). Expression of claudin-2 was up-regulated in CLE^+^ patients compared with CLE− patients or controls (*p* = 0.023). CLE^+^ patients had lower occludin levels in duodenal samples vs. controls (*p* = 0.022). Eosinophil degranulation was increased and ECP levels were higher in CLE^+^ patients than controls (*p* = 0.03).	More than 50% of patients with IBS could have nonclassical food allergy, with immediate disruption of the intestinal barrier upon exposure to food antigens. Duodenal tissues had immediate increases in the expression of claudin-2 and decreases in occludin. The increased eosinophil degranulation in CLE+ indicates an atypical food allergy.

CD: Celiac disease; DPBC-C: double-blind placebo-controlled challenge; CLE+: patients with a positive reaction to CLE antigen exposure; CLE−: patients with a negative reaction to CLE antigen exposure; ECP: eosinophil cationic protein; HCs: healthy controls; IEL: intraepithelial lymphocytes; KIRs: killer immunoglobulin-like receptors; NCWS: non-celiac wheat sensitivity; PCR-SSOP: polymerase chain reaction–sequence-specific oligonucleotide probe; CLE: confocal laser endomicroscopy.

**Table 2:** Studies regarding the clinical features of NCGWS from 2019 to 2024.

Title	Authors	Type of Study	Population	Diagnostic Criteria for NCGWS	Methods	Results	Author’s Conclusions
Dietary and symptom assessment in adults with self-reported non-coeliac gluten sensitivity	Skodje G.I. et al., 2019 [26]	Cross-sectional study.	65 NCGWS subjects. Mean age = 44 years. *N* = 57 (88%) were female, *n* = 8 male.	Self-reported	Baseline characteristics of NCGWS on GFD were recorded. Dietary intake was obtained by a seven-day food record Symptoms were assessed using VAS, GSRS-IBS, and HR-QoL.	Mean proportions of energy were 43% from fat, 40% from carbohydrate and 17% from protein. Intakes of vitamin D, folic acid, calcium, iron, and iodine were lower than recommended, mean (SD) intake of FODMAPs was 11.6 g (8.7). GI symptoms as scored by 100 mm (VAS) were all below 15 mm, wind and bloating were the most expressed. Tiredness, concentration difficulties, fatigue, and muscle/joint pain were scored highest among extra-intestinal symptoms. GSRS-IBS scores were correlated with mild depression (r = 0.43) and inversely correlated with five sub-domains of HR-QoL (−0.29 < r < −0.26).	Subjects with self-reported NCGWS had high proportion of energy from fat and sub-optimal intakes of several micronutrients. Subjects reported GI and extra-intestinal symptoms and reduced HR-QoL. Importance of dietary education and nutritional follow-up of subjects on GFD must be highlighted
Symptoms of gluten ingestion in patients with non-celiac gluten sensitivity: A randomized clinical trial	Moleski S.M. et al., 2021 [14]	Prospective, randomized, double-blinded crossover trial.	30 NCGWS and 43 HCs were placed on a GFD.	Self-reported	Patients received 0.5 or 2 g/day of gluten for 7 days each and placebo for a total of 4 weeks. Symptoms were evaluated using the CSI. Urine and stool samples were collected for the detection of GIPs.	No difference in symptom severity within the NCGWS group whether receiving placebo or gluten (32.69 vs. 31.54, *p* = 0.64). NCGWS had significantly higher CSI scores at baseline than healthy controls. Patients with NCGWS were less likely to have stool and urine GIPs than healthy patients.	Patients with NCGWS were more adherent to the GFD Patients with NCGWS had increased symptom severity at baseline compared with healthy controls. Neither group had significantly increased symptoms after ingestion of gluten.
Evaluation of Non-Celiac Gluten Sensitivity in Patients with Previous Diagnosis of Irritable Bowel Syndrome: A Randomized Double-Blind Placebo-Controlled Crossover Trial	Barone M. et al., 2020 [17]	Randomized Double-Blind Placebo-Controlled Crossover Trial.	30 patients *n* = 23 female, *n* = 7 male, aged 42.2 ± 12.5 years. A total of 26 patients followed a low FODMAPs-GFD and were exposed to the gluten/placebo challenge.	DPBC-C	All subjects received a low FODMAP diet that was also gluten-free (low FODMAP-GFD). Patients presenting an improvement of symptoms were exposed to gluten or placebo. The response to dietary treatments was evaluated by VAS.	26/30 patients benefited from the administration of low FODMAP-GFD and were exposed to the gluten/placebo challenge. After the challenge 46.1% of the patients were NCGWS+. This percentage became only 19.2% using a different method (mean ∆-VAS score plus two Standard Deviations).	FODMAP intolerance could hide the response to a challenge test with gluten for the identification of NCGWS in IBS patients. A low FODMAP-GFD followed by gluten/placebo challenge can identify patients with NCGWS better.
Effects of fructan and gluten on gut microbiota in individuals with self-reported non-celiac gluten/wheat sensitivity-a randomised controlled crossover trial	Herfindal AM. et al., 2024 [22]	Randomized controlled crossover trial.	59 participants with self-reported NCGWS.	Self-reported	Participants underwent three different 7-day diet challenges with gluten (5.7 g/day), FOS-fructans (2.1 g/day), and placebo separately.	After the FOS-fructans challenge, *Fusicatenibacter* increased, while *Eubacterium (E.) coprostanoligenes* group, *Anaerotruncus*, and *unknown Ruminococcaceae* genera decreased. The gluten challenge was characterized by increased abundance of *Eubacterium xylanophilum* group. No differences were found for α-diversity, overall bacterial community structure (β-diversity), fecal metabolites (SCFA), or NGAL/LCN2. GI symptoms in response to FOS-fructans were not linked to shifts in the gut bacterial community. The reduction in *E. coprostanoligenes* group following the FOS-fructans challenge was associated with increased GI pain. Changes in GI symptoms following the FOS-fructans and gluten challenges were linked to varying bacterial abundances at baseline.	FOS-fructans induced more GI symptoms than gluten in the NCGWS patients. No substantial shifts in the composition nor function of the fecal microbiota could explain the differences found. Individual variations in baseline bacterial composition/function may influence the GI symptom response. The change in *E. coprostanoligenes* group could suggest further evaluation in the involvement of bacterial species on GI symptoms.
Does a Gluten-Free Diet Improve Quality of Life and Sleep in Patients with Non-Coeliac Gluten/Wheat Sensitivity?	Cotton C, et al., 2023 [31]	Prospective observational study.	NCGWS = 114 (84.8% female), median age 46 years (IQR: 35–59). CD = 170 (71.2% female), median age 52 years (IQR: 37–61).	Self-reported	Patients with NCGWS completed the CDAT, CSI and SCI. A second group of patients with CD completed the CDAT questionnaire only. Results were compared with the CDAT responses from the NCGWS group.	For the NCGWS cohort (*n* = 125), the median CDAT score was 17/35, indicating poor adherence. The median CSI score was 44/80, with 40% associated with a poor QoL. The median SCI score was 14/32 DSM-V criteria for insomnia was met by 42% of patients. Positive correlation between CSI and CDAT scores (r = 0.59, *p* < 0.0001) Negative correlation between SCI and CDAT scores. In the CD cohort (*n* = 170), median CDAT score was 13/35. Patients with NCGWS had poorer adherence compared with CD.	Patients with NCGWS adhere to a GFD less than those with CD. Poorer adherence to a GFD in patients with NCGWS correlates with a worse QoL and sleep performance.
Prevalence of Non-Celiac Gluten Sensitivity in Patients with Refractory Functional Dyspepsia: a Randomized Double-blind Placebo Controlled Trial	Shahbazkhani B. et al., 2020 [18]	Randomized double-blind placebo-controlled crossover trial	77 patients with RFD A total of 27 patients (*n* = 11 male, *n* = 16 female) entered the DPBC-C.	DPBC-C	27 patients responded to a GFD and were randomly divided into two group A and B and entered the DPBC-C arm. VAS was used to assess their GI and extra-GI symptoms. After the DPBC-C phase, all the patients were followed for 3 more months to evaluate symptoms recurrence during gluten re-introduction. Patients reporting symptoms recurrence were diagnosed as NCGWS. The symptomatic response was defined as a variation of at least 30% from the baseline.	Out of 77 patients with RFD, 50 (65%) did not respond to GFD. 27 (35%) cases showed GI symptoms improvement while on the GFD. Symptoms recurred in 5 cases (6.4% of patients with RFD, 18% of GFD responders) after blind gluten ingestion. Extra-intestinal symptoms as fatigue and weakness (*p* = 0.000), musculo-skeletal pain (*p* = 0.000) and headache (*p* = 0.002) improved in NCGWS patients on GFD.	NCGWS is highly prevalent among patients with RFD therefore evaluating the effect of GFD in patients with RFD seems a reasonable approach.
Wheat Sensitivity and Functional Dyspepsia: A Pilot, Double-Blind, Randomized, Placebo-Controlled Dietary Crossover Trial with Novel Challenge Protocol	Potter M. D. E. et al., 2020 [20]	Pilot randomized double-blind, placebo controlled, dietary crossover trial.	11 participants with Rome III criteria FD; 75% female, 25% male. Mean age 43 years.	DPBC-C	Patients were individually counselled on a diet low in both gluten and FODMAPs for four weeks (elimination diet phase). Those who had a >30% response to the run-in diet, as measured by the NDI, were rechallenged with ‘muesli’ bars containing either gluten, fructans, or placebo in randomized order. Those with symptoms which significantly reduced during the elimination diet, but reliably reappeared (with gluten or fructans rechallenge were deemed to have wheat-induced FD.	Nine participants completed the elimination diet phase, four qualified for the rechallenge phase. The low FODMAPs-GFD led to an overall (albeit non-significant) improvement in symptoms of FD in the diet elimination phase	A specific trigger could not be identified. Larger trials are required to determine whether specific components of wheat induce symptoms in FD.
Effect of a Gluten-Free Diet on Whole Gut Transit Time in Celiac Disease (CD) and Non-Celiac Gluten Sensitivity (NCGS) Patients: A Study Using the Wireless Motility Capsule (WMC)	Cobos-Quevedo O. et al., 2024 [34]	Prospective study.	CD *n* = 12; NCGWS *n* = 12	DPBC-C	WMC was used to assess regional (measurements of gastric, small bowel, and colonic transit times) and whole gut transit. Patients underwent evaluations at baseline and 4 weeks after having a GFD.	At baseline CD patients exhibited prolonged colonic and intestinal transit times when compared with those with NCGWS (*p* < 0.05). After 4-week GFD, CD patients experienced significant reductions in intestinal and colonic transit times, and small intestine contractility. NCGWS individuals showed improvements in intestinal transit time and contractility with a GFD The GFD did not significantly impact intragastric, intestinal, or colonic pH.	GFD has some positive effects on intestinal and colonic transit and contractility in CD patients, and to a lesser extent, in those with NCGWS.
Anemia in non-celiac wheat sensitivity: Prevalence and associated clinical and laboratory features	Mansueto P et al., 2023 [29]	Retrospective analysis plus a perspective analysis.	244 NCWS, 2 control groups CD and IBS 31 NCWS anemic patients were prospectively re-evaluated after at least 12 months on WFD.	DPBC-C	Retrospective analysis: data from NCGWS patients were reviewed and compared with CD and IBS Associated autoimmune diseases and coexisting allergies, systemic nickel allergy syndrome, and allergic rhino-conjunctivitis/asthma/atopic dermatitis) HLA DQ2/DQ8 haplotypes and serum ANA were assessed. Prospective part: NCWS patients with anemia at diagnosis were contacted and those who had been following a strict WFD for at least 12 months had been selected. Patients repeated a complete blood and reticulocyte count, serum ferritin, vitamin B12, and folic acid level analysis. Treatment with iron, vitamin B12 and folate, was prescribed during the prospective phase of the study, if considered appropriate.	Anemia prevalence in NCWS patients was 34.8% (mean hemoglobin 10.4 ± 1.4 g/dL), significantly higher than in IBS (17.4%, *p* = 0.03), but not in CD ones. The NCWS group had sideropenic-like features Both anemia prevalence and sideropenic-like features were more evident in CD than in NCWS patients Significant differences were found in anemic vs. non-anemic NCWS patients as regards to female sex, diagnostic delay, poly/hypermenorrhea, iron deficiency, and higher TSH values. A long-term WFD significantly reduced anemia and improved iron metabolism.	Microcytic/hypochromic anemia and altered iron metabolism occur frequently in NCWS and can be treated with a long-term strict WFD. NCWS should be included in differential diagnosis of anemic patients with “functional gastrointestinal troubles”.

ANAs: Anti-nuclear antibodies; CD: celiac disease; CDAT: Celiac Disease Adherence Test; CSI: Celiac Symptom Index; DPBC-C: double-blind placebo-controlled challenge; DSM: Diagnostic and Statistical Manual; FD: functional dyspepsia; FODMAPs: fermentable oligosaccharides, disaccharides, monosaccharides, and polyols; FOSs: fructo-oligosaccharides; GFD: gluten-free diet; GI: gastrointestinal; GIPs: gluten immunogenic peptides; GSRS-IBS: Gastrointestinal Symptoms Rating Scale-Irritable Bowel Syndrome; HCs: healthy controls; HR-QoL: health-related quality of life; IQR: interquartile range; IBS: irritable bowel syndrome; NCGS: non-celiac gluten sensitivity; NCGWS: non-celiac gluten/wheat sensitivity; NCWS: non-celiac wheat sensitivity; NDI: Nepean Dyspepsia Index; NGAL/LCN2: neutrophil gelatinase-associated lipocalin 2; SCFA: short-chain fatty acid; VAS: visual analog scale; RFD: refractory functional dyspepsia; SCI: Sleep Condition Indicator; WFD: wheat-free diet; WMC: wireless motility and pH capsule.

**Table 3:** Studies analyzing new diagnostic tools for NCGWS from 2019 to 2024.

Title	Authors	Type of Study	Population	Diagnostic Criteria for NCGWS	Methods	Results	Author’s Conclusions
Serum zonulin and its diagnostic performance in non-coeliac gluten sensitivity	Barbaro MR. et al., 2020 [32]	Multicenter prospective study.	86 patients with self-reported or double-blind confirmed NCGS, 59 patients with IBS-D, 15 patients with CD and 25 HCs	DPBC-C	Zonulin serum levels, clinical and symptomatic data were assessed. The effect of diet on zonulin levels was evaluated in a subgroup of patients with NCGS.	NCGS and CD patients had significantly increased levels of zonulin compared with HCs Self-reported NCGS showed increased zonulin levels compared with double-blind confirmed and not-confirmed NCGS. Six-month wheat avoidance significantly reduced zonulin levels only in HLA-DQ2/8-positive participants with NCGS. The diagnostic accuracy of zonulin levels in distinguishing NCGS from IBS-D was 81%. A diagnostic algorithm combining zonulin levels, symptoms and gender could improve the accuracy up to 89%.	Zonulin can be considered a diagnostic biomarker in NCGS. The reduction in zonulin levels was seen only in NCGS carrying the HLA-DQ2/8 genotype after a WFD.
Prospective, double-blind diagnostic multicenter study of confocal laser endomicroscopy for wheat sensitivity in patients with irritable bowel syndrome	Bojarski C. et al., 2022 [27]	Prospective, double-blind multicenter study.	130 patients fulfilling the Rome III criteria for IBS	DPBC-C	Patients underwent CLE after wheat (index test), soy, yeast, or milk exposure. Patients with IBS responding to 2 months of GFD were classified as having wheat sensitivity. After 2 months, CLE results were unblinded and patients were advised to exclude those food components that had led to a positive CLE reaction. The clinical response was assessed at follow-up after 6 and 12 months.	56.9% of patients were considered wheat-sensitive. 38 of 74 patients were correctly identified by CLE (sensitivity 51.4%; 97.5% CI: 38.7% to 63.9%). A total of 38 of 56 patients without wheat sensitivity were correctly identified by CLE (specificity 67.9%; 97.5% CI: 52.9% to 79.9%). At 6 months, CLE identified 49 of 59 food-sensitive patients (sensitivity 83.1%; 97.5% CI: 69.9% to 91.3%). Specificity was only 32% (97.5% CI: 15.7% to 54.3%).	CLE is an invasive procedure, and its diagnostic accuracy is too low to recommend the widespread use of this procedure.
Fecal calprotectin levels in patients with non-celiac wheat sensitivity: a proof of concept	Seidita A. et al., 2024 [28]	Prospective, double-blind multicenter study.	201 NCWS and 50 IBS/FD patients were found eligible and then recruited	DPBC-C	FCP was analyzed to describe its role as a NCWS diagnostic biomarker before and after a WFD.	31.3% NCWS patients had above normal FCP values (NCWS FCP+). With a cut-off value > 41 µg/g, FCP showed a 58.6% sensitivity and a 98.0% specificity (AUC 0.755, 95% CI 0.702–0.837) in distinguishing NCWS from IBS/FD patients. Of the 63 NCWS FCP+, 65.1% had negative FCP values after ≥6 months of WFD, with a significant reduction in FCP values (*p* < 0.0001). All NCWS FCP- subjects still preserved negative FCP values after ≥6 months of WFD. Strict WFD adherence reduced FCP values, normalizing them in 65.1% of NCWS FCP + subjects.	FCP can be a useful diagnostic marker for differentiating between NCWS and IBS/FD as a supplementary tool. Two NCWS subgroups might exist: NCWS FCP+ characterized by a predominant inflammatory/immunologic pattern and NCWS FCP-featuring non-immuno-mediated pathogenetic mechanisms.
Sieving out non-celiac gluten sensitivity amongst patients with irritable bowel syndrome	Ahmed et al., 2024 [33]	Prospective study	492 patients with IBS	DPBC-C	Patients with positive AGAs (IgA and/or IgG) were asked to follow a GFD for 6 weeks. Responders were given gluten rechallenge. Diagnosis of NCGS was confirmed with symptom recurrence on a gluten containing diet.	Of 492 patients with IBS, AGA was positive in 61 (12.4%) patients. Of 31 patients following a GFD, 17 (54.8%) had complete (>30% improvement) and 10 (32.2%) had partial (>20% improvement) responses. After gluten challenge, all of the responders developed symptoms. AGA levels decreased significantly in almost all GFD-responders.	12.4% of IBS patients have biological evidence of gluten/wheat-sensitivity. Almost 87% of patients with IBS had AGAs respond to GFD. AGA may be explored as a biomarker for NCGS.

AGAs: Anti-gliadin antibodies; AUC: area under the curve; CD: celiac disease; CI: confidential interval; CLE: confocal laser endomicroscopy; DPBC-C: double-blind placebo-controlled challenge; FCP: fecal calprotectin; FD: functional dyspepsia; FODMAPs: fermentable oligosaccharides, disaccharides, monosaccharides, and polyols; GFD: gluten-free diet; GIPs: gluten immunogenic peptides; HCs: healthy controls; HLA: human leukocyte antigen; IBS: irritable bowel syndrome; IBS-D: diarrhea-predominant IBS; NCGS: non-celiac gluten sensitivity; NCGWS: non-celiac gluten/wheat sensitivity; NCWS: non-celiac wheat sensitivity; WFD: wheat-free diet.

**Table 4:** Studies regarding dietary interventions for NCGWS from 2019 to 2024.

Title	Authors	Type of Study	Population	Diagnostic Criteria for NCGWS	Methods	Results	Author’s Conclusions
Effect of Gluten Ingestion and FODMAP Restriction on Intestinal Epithelial Integrity in Patients with Irritable Bowel Syndrome and Self-Reported Non-Coeliac Gluten Sensitivity	Ajamian M. et al., 2021 [15]	Placebo-controlled, randomized, double-blind crossover intervention	37 IBS subjects, mean age = 45 years. *N* = 31 female, *n* = 6 male. 49 HC, mean age = 39 years. *N* = 32 female, *n* = 17 male	Self-reported	Participants remained on a GFD, normal FODMAPs diet, and their intake and symptoms were documented for 1 week. In the 2-week run-in period they reduced their FODMAPs intake. Patients then received either low gluten, high gluten, and placebo diet on a background low FODMAPs diet for one week followed by a minimum 2-week wash-out before the second diet was commenced for 1 week. The same process was followed for the third diet. Blood levels of syndecan-1 and intestinal fatty acid-binding protein, lipopolysaccharide-binding protein and soluble CD14 were measured.	In 33 patients, only syndecan-1 concentrations during their habitual diet were elevated (median 43 ng mL^−1^) compared with 23 ng mL^−1^ in 49 healthy subjects (*p* < 0.001). On a low FODMAPs diet, symptoms reduced and levels of syndecan-1 (but not of other markers) fell by a median 3335% (*p* < 0.001) irrespective of whether gluten is present or not.	Gluten ingestion has no effect on epithelial integrity or symptoms. Reducing FODMAPs intake reduces both symptoms and reverses apparent colonic epithelial injury. Self-reported gluten sensitivity population is heterogeneous.
Exposure to Different Amounts of Dietary Gluten in Patients with Non-Celiac Gluten Sensitivity (NCGWS): An Exploratory Study	Roncoroni L. et al., 2019 [30]	Interventional trial	24 NCGWS patients	DPBC-C	Patients followed a GFD for 3 weeks, then were exposed to an incremental quantity of gluten. Three groups were compared at baseline and immediately after the return of symptomatology: (i) a group tolerating a low-gluten diet (3.5 g gluten/day, week 1, *n* = 8), (ii) a group tolerating a mid-gluten diet (8 g gluten/day, week 2, *n* = 6), and (iii) a group tolerating a high-gluten diet (13 g gluten/day, week 3, *n* = 8). GI symptoms and QoL after the re-introduction of gluten were evaluated.	Constipation (46%), abdominal pain (50%) and dyspepsia (38%) were the most reported symptoms. A decrease in several SF-36 sub-scores (all *p* < 0.03) after gluten re-introduction was only observed in the group tolerating the low-gluten diet. The same group showed a lower post-intervention role-emotional SF-36 score (*p* = 0.01). Only in the group tolerating a low-gluten diet (*p* = 0.01) a decrease in the general perception of well-being was found.	Different responses from patients with NCGWS were observed after the re-introduction of gluten. A group of patients scarcely tolerated gluten reintroduction affecting the QoL and general well-being of a group of patients. Another subgroup could tolerate higher doses of gluten.
A Durum Wheat Variety-Based Product Is Effective in Reducing Symptoms in Patients with Non-Celiac Gluten Sensitivity: A Double-Blind Randomized Cross-Over Trial	Ianiro G. et al., 2019 [19]	Randomized double-blind placebo-controlled crossover trial	42 NCGWS patients, 70.6% females. A total of 34 patients completed the study	DPBC-C	Enrolled subjects were assigned to: (A) a two-week diet with Senatore Cappelli wheat variety pasta; (B) a two-week diet with standard commercial pasta. After two weeks on a GFD, each patient crossed over to the other treatment group. Symptoms were assessed with a modified version of the GSRS.	Patients reported lower symptoms scores (both GI and extra GI) after eating Senatore Cappelli pasta than standard pasta (*p* = 0.03).	Patients with NCGWS experienced lower GI and extra-intestinal symptom scores after eating the Senatore Cappelli wheat variety compared with a standard wheat. New dietary alternatives may be available to patients with NCGWS.
No Difference in Tolerance between Wheat and Spelt Bread in Patients with Suspected Non-Celiac Wheat Sensitivity	Zimmermann J. et al., 2022 [24]	Single-blind randomized trial	24 NCWS patients with suspected spelt tolerance	Self-reported	Six different study breads were used in this crossover challenge, each at 300 g per day for 4 days followed by a wash-out phase of 3 days. Spelt and wheat breads were used, either baked with a traditional (T) or a current (C) recipe. A gluten-free bread with 1.5% added oligosaccharides (+FODMAPs) and a gluten-free bread with 5% added wheat gluten (+Gluten) were also used. IBS-SSS was used to assess symptoms.	IBS-SSS was higher than self-estimated by the participants after spelt bread consumption and lower for wheat bread resulting in no difference between wheat and spelt bread tolerance. The +FODMAP bread was better tolerated than both T breads (*p* = 0.003 for spelt; *p* = 0.068 for wheat) Neither signs of inflammation nor markers for intestinal barrier integrity were influenced.	Differences in expected symptoms resulting from wheat and spelt products cannot be confirmed with these data. This suggests the presence of nocebo effect for wheat and a placebo effect for spelt.
Two randomized crossover multicenter studies investigating gastrointestinal symptoms after bread consumption in individuals with noncoeliac wheat sensitivity: do wheat species and fermentation type matter?	De Graaf M. C. G. et al., 2024 [23]	Randomized double-blind placebo-controlled crossover multicenter trial.	20 NCGWS patients in study A, 20 NCGWS patients in study B	Self-reported	NCWS patients received 5 slices of YF (study A, *n* = 20) or sourdough fermented SF (study B, *n* = 20) bread made of bread wheat, spelt, or emmer in a randomized order on 3 separate test days. A run-in period of 3 d of a symptom-free diet and a wash-out period of ≥7 d preceded every test day. GI symptoms were evaluated ΔVAS. Responders were defined as an increase in ΔVAS of ≥15 mm for overall GI symptoms.	GI symptoms did not differ significantly between breads of different grains. The number of responders was also comparable for both YF (6 to wheat, 5 to spelt, and 7 to emmer, *p* = 0.761) and SF breads (9 to wheat, 7 to spelt, and 8 to emmer, *p* = 0.761).	The majority of NCWS individuals experienced some GI symptoms for more than one of the breads. No differences were found between different grains for either YF or SF breads.
The effect of expectancy versus actual gluten intake on gastrointestinal and extra-intestinal symptoms in non-coeliac gluten sensitivity: a randomised, double-blind, placebo-controlled, international, multicentre study	De Graaf M. C. G. et al., 2024 [21]	A randomized, double-blind, placebo-controlled, international, multicenter study	83 NCGWS. *N* = 71 (86%) female, *n* = 12 (14%) = men	Self-reported	Patients were randomly assigned to E+G+ (*n* = 21), E+G− (*n* = 21), E-G+ (*n* = 20), or E-G− (*n* = 22) (see below). Participants had to follow a gluten-free or gluten-restricted diet for at least 1 week before (and throughout) study participation and had to be asymptomatic or mildly symptomatic (overall GI symptom score ≤ 30 mm on VAS) while on the diet. Overall GI symptom scores were measured using VAS, before breakfast and hourly for 8 h.	Mean overall GI symptom score was significantly higher for E+G+ than for E-G+ but not for E+G−. There was no difference between E+G− and E-G+ E+G− and E-G− and E-G+ and E-G. Adverse events were reported by two participants in the E+G− group (itching jaw [*n* = 1]; feeling lightheaded and stomach rumbling [*n* = 1]) and one participant in the E-G+ group (vomiting).	The largest effect on GI symptoms was given by the combination of expectancy and actual gluten intake, reflecting a nocebo effect, although an additional effect of gluten cannot be ruled out. A possible involvement of the gut-brain interaction in NCGWS can be suggested.

CI: Confidential interval; DPBC-C: double-blind placebo-controlled challenge; FODMAPs: fermentable oligosaccharides, disaccharides, monosaccharides, and polyols; GFD: gluten-free diet; GI: gastrointestinal; GSRS: Gastrointestinal Symptom Rating Scale; HCs: healthy controls; IBS: irritable bowel syndrome; IBS-SSS: IBS Severity Scoring System; SF-36: Short Form health survey-36; NCGWS: non-celiac gluten/wheat sensitivity; NCWS: non-celiac wheat sensitivity; SF: sourdough fermented; QoL: quality of life; VAS: visual analog scale; WFD: wheat-free diet; YF: yeast fermented.

**Table 5:** Details of the study regarding therapeutic strategies.

Title	Authors	Type of Study	Population	Diagnostic Criteria for NCGWS	Methods	Results	Author’s Conclusions
Use of a proline-specific endopeptidase to reintroduce gluten in patients with non-coeliac gluten sensitivity: A randomized trial	Scricciolo A. et al., 2022 [13]	Randomized, double-blind, placebo-controlled monocentric study.	23 patients with NCGWS who were allocated to a placebo group (*n* = 11, age 38.4 ± 2.9 years) or an intervention group (*n* = 12, age 39.5 ± 3.1 years).	DPBC-C	NCGWS patients were asked to take P1016 or placebo during gluten reintroduction. Symptoms were evaluated with VAS, SF-36, and SCL-90 on a weekly basis. Over a 3-week period gluten was reintroduced gradually under nutritional control.	P1016 failed to show a improvement on symptoms. During gluten reintroduction, patients reported a significant increase in abdominal pain and a worsening of stool consistency. The two groups did not differ in their average SF-36 and SCL-90 scores.	P1016 showed a lack of efficacy in the management of NCGWS patients and the possible reintroduction of gluten.

DPBC-C: Double-blind placebo-controlled challenge; NCGWS: non-celiac gluten/wheat sensitivity; SCL-90: Symptom CheckList-90; SF-36: Short Form health survey-36; VAS: visual analog scale.

## Data Availability

No new data were created or analyzed in this study. Data sharing is not applicable to this article.

## Abbreviations

The following abbreviations are used in this manuscript:

ATIs	Amylase trypsin inhibitor
AGAs	Anti-gliadin antibodies
AUC	Area under the curve
CI	Confidential interval
CLE	Confocal laser endomicroscopy
CD	Celiac disease
DGBI	Disorders of the gut–brain interaction
DPBC-C	Double blind placebo-controlled challenge
ECP	Eosinophil cationic protein
FCP	Fecal calprotectin
FD	Functional dyspepsia
FODMAPs	Fermentable oligosaccharides, disaccharides, monosaccharides, and polyols
FS	Fermented sourdough
FY	Fermented yeast
GFD	Gluten-free diet
GI	Gastrointestinal
GIPs	Gluten immunogenic peptides
GSRS	Gastrointestinal symptom rating scale
HCs	Healthy controls
HLA	Human leukocyte antigen
HR-QoL	Health-related quality of life
IBS	Irritable bowel syndrome
IBS-D	Diarrhea-predominant IBS
IBS-SSS	IBS Severity Scoring System
IEL	Intraepithelial lymphocytes
IQR	Interquartile range
KIRs	Killer immunoglobulin-like receptors
NCGS	Non-celiac gluten sensitivity
NCWS	Non-celiac wheat sensitivity
NCGWS	Non-celiac gluten/wheat sensitivity
NDI	Nepean Dyspepsia Index
NGAL/LCN2	Neutrophil gelatinase-associated lipocalin 2
QoL	Quality of life
RCT	Randomized controlled trial
RFD	Refractory functional dyspepsia
SCFA	Short-chain fatty acid
SCI	Sleep condition indicator
SCL-90	Symptom CheckList-90
SF-36	Short Form health survey-36
VAS	Visual analog scale
WA	Wheat allergy
WFD	Wheat-free diet
WGA	Wheat germ agglutinins
WMC	Wireless motility and pH capsule
